# Dietary Application of Grape (*Vitis Vinifera* L.) Pomace for Fattening Lambs: Implications on Growth Performance, Rumen Fermentation, and Meat Fatty Acid Composition

**DOI:** 10.1002/vms3.70495

**Published:** 2025-08-04

**Authors:** Moslem Bashtani, Mahdi Badiee Baghsiyah, Seyyed Homayoun Farhangfar, Navid Ghavipanje

**Affiliations:** ^1^ Department of Animal Science, Faculty of Agriculture University of Birjand Birjand Iran; ^2^ South Khorasan Agricultural and Natural Resources Research and Education Center Agricultural Research, Education and Extension Organization (AREEO) Birjand Iran

**Keywords:** baluchi lambs, blood metabolites, grape by‐products, meat, ruminal fermentation, sustainability

## Abstract

Dietary inclusion of grape pomace (GP) for finishing lambs is expected to improve performance and product quality, thanks to its rich polyphenolic content and favourable fatty acid (FA) profile. This study determines the implications of feeding varying levels of GP (*Vitis vinifera L*. cultivar SiahGohar) on production performance, rumen fermentation, blood parameters, and meat FA composition in finishing lambs. Twenty‐one Baluchi lambs [28.35 ± 1.54 kg of initial body weight (IBW), mean ± standard deviation (SD)] were randomly assigned to four diets containing 0, 6%, 12%, and 18% g GP [dry matter (DM) basis] in total mixed rations (TMR). The trial last for 90 days, preceded by 21 days of adaptation. Results showed that the dietary inclusion of GP did not affect the DMI (*p* = 0.8452) of fattening lambs, while it improved the final body weight (FBW, *p* = 0.027), average daily gain (ADG, *p* = 0.016), and feed to gain ratio (F:G, *p* = 0.001). Lambs fed on GP diets tended to increase ruminal pH (*p* = 0.100); however, NH_3_‐N levels remained unaffected (*p* = 0.160). There was no diet effect (*p* > 0.5) on plasma concentrations of glucose, triglyceride, cholesterol, albumin, creatinine, BUN, aspartate aminotransferase (AST), and alanine aminotransferase (ALT). Dietary inclusion of GP also led to increased muscle accumulation of C18:2 n‐6 (*p < 0.0001*), C18:3 n‐3 (*p = 0.005*), C20:3 n‐3 (*p < 0.0001*), total MUFA (*p < 0.0001*), and PUFA (*p < 0.0001*). GP also mitigated lipid oxidation in lamb's meat, as indicated by lower (*p < 0.0001*) malondialdehyde (MDA) levels. Further investigation is warranted to verify the potential effects of GP on the rumen microbiome, colour stability, and shelf‐life of meat, as well as to elucidate the mechanisms underlying the action of polyphenols and their composition contained in GP.

## Graphical abstract

Dietary grape pomace (6–18% DM) enhances lamb growth, improves ruminal fermentation, and increases muscle accumulation of health‐promoting FA, including 18:2 n‐6, 18:2 c9t11, C18:3 n‐3, C20:3 n‐3, and total PUFA

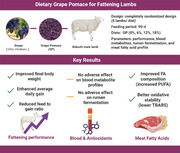

## 1 Introduction

The global human population is projected to surpass 10 billion by 2050, driving a 59–98% increase in food demand and a 70% expansion in livestock population (Jalal et al. 2023). However, livestock production faces occasional shocks, including global warming (Felini et al. 2024; Yang et al. 2021), feed shortages and high feed costs (Gasparini et al. 2024), food‐feed‐fuel competition, and environmental impacts (Vastolo et al. 2024; Correddu et al. 2023). In this context, unconventional feed resources have emerged as a solution for sustainable livestock production, reducing costs and supporting a circular economy (Yang et al. 2021). Globally, an increase in agri‐food commodity production generates substantial by‐products, often discarded in landfills or rivers, leading to environmental contamination (Correddu et al. 2023). Repurposing these by‐products as animal feed offers a promising strategy to enhance sustainability and economic viability in ruminant systems while favouring product quality and ensuring (Siller‐Sánchez et al. 2024; Yang et al. 2021; Jalal et al. 2023; Correddu et al. 2023).

Grape (*Vitis vinifera* L.), a major global fruit crop, yields approximately 75 million tonnes annually, with China, Italy, Spain, and France as leading producers (FAO 2025). The grape pomace (GP), a by‐product of wine and juice production, constitutes ∼25% of pressed grapes, comprising skin (∼51%), seeds (∼47%), and stalks (∼2) (Antonić et al. 2020). Containing bioactive compounds, such as phenolic acids, flavonoids, and tannins, GP exhibits antioxidant, antimicrobial, antiviral, antiaging, antiallergic, and anticancer properties (Siller‐Sánchez et al. 2024; Machado et al. 2024). Although GP is not hazardous, improper management can cause environmental issues, including crop phytotoxicity, water pollution, oxygen depletion from tannins, and pest attraction, necessitating its transformation into value‐added products like animal feed (Siller‐Sánchez et al. 2024). In recent years, researchers have paid great attention to incorporating GP as animal feed.

As reviewed by Antonić et al. (2020), GP contains 3.57‐14.17% crude protein (CP), 1.73‐9.10% ash, 1.14‐13.9% ether extract (EE), and 0.28‐8.70% total polyphenolic content [dry matter (DM) basis]. Its lipid content (4.62% to 12.5%) is rich in unsaturated fatty acids (UFA), linoleic acid (59.0–70.9%), oleic acid (15.5–21.8%), and α‐linolenic acid (1.05–2.85%) (Antonić et al. 2020; Blasi et al. 2024), aligning with consumer demands for a healthier FA profile. As a substitute for conventional feed ingredients like beet pulp (BP), GP offers comparable CP (13.2% vs. 9.2% for BP) and neutral detergent fibre (NDF, 50% vs. 42.2% for BP) (Mirzaei‐Aghsaghali and Maheri‐Sis 2008), with the added benefit of bioactive compounds that may enhance animal health and product quality (Antonić et al. 2020). However, high lignin and polyphenol levels, particularly proanthocyanidins, may reduce digestibility by inhibiting digestive enzymes, thereby limiting GP's use as feed (Blasi et al. 2024). Currently, only 3% of global GP is used as animal feed, highlighting an opportunity for further exploration (Zhang et al. 2017).

In ruminants, dietary GP has demonstrated immune‐modulatory effects without negatively affecting milk production in ewes (Nudda et al. 2015). Furthermore, it has been well established that the bioactive compounds presented in GP can also enhance the quality of milk and meat (Blasi et al. 2024; Correddu et al. 2023). Dietary supplementation of lactating dairy cows with 10% GP has been shown (Corereddu et al., 2015) to effectively increase the concentrations of vaccenic acid (C18:1 trans‐11) and linoleic acid (C18:2 n‐6) in milk. In lambs, diets containing 12.2% GP improved average daily gain (ADG) and feed efficiency without compromising meat quality (Chikwanha et al. 2019). Whereas feeding diets with 10% GP showed increased ADG and enhanced enzymatic antioxidant activity (Zhao et al. 2018).

While previous studies have investigated GP in ruminants (i.e., Chikwanha et al. 2019a; Zhao et al. 2018; Molosse et al. 2023; Massaro et al. 2021), they have primarily focused on productivity parameters, leaving gaps in our understanding of the underlying mechanisms and comprehensive assessment of product quality. Specifically, the impact of GP on performance, antioxidant activity, meat quality, and FA profiles in feedlot lambs remains equivocal. This study addresses these gaps by evaluating the effects of varying GP levels on growth performance, antioxidant activity, rumen fermentation, and meat FA composition in Baluchi fattening lambs. It is hypothesised that GP can replace conventional feed ingredients, such as sugar beet, in lamb diets, leveraging its bioactive compounds to enhance growth performance and shift meat FA profiles toward a healthier composition.

## 2 Materials and Methods

### 2.1 Ethics Statement

The experiment was carried out according to the guidelines of the Iranian Council of Animal Care (1995). All experimental protocols and implemented procedures were reviewed by the Animal Welfare and Ethical Review Board of the Department of Animal Science, University of Birjand. This experiment was performed at the experimental farm of the Faculty of Agriculture, University of Birjand, Iran (longitude and latitude, 37.42°N and 57.31°E, 1491 m above sea level, and an annual average rainfall of 171 mm) from August 2019 to December 2019.

### 2.2 Animals, Diets and Measurements

Twenty‐four Baluchi male lambs with an initial body weight (IBW) of 28.35 ± 1.54 kg (mean ± standard deviation) were allocated to four dietary treatments (6 lambs/diet) in a completely randomised design (CRD) for a feeding period of 90 days preceded by a 21‐day adaptation period. Prior to the start of the trial, all lambs were weighed and dewormed with ivermectin (Iversheep, Rooyan, Tehran, Iran) at a dose of 0.1 mL per 5 kg of live weight. A vitamin supplement (AD3EC Rooyan, Rooyan, Tehran, Iran) was administered intramuscularly. The experimental diets were (1) control, with no GP (Ctrl); (2) GP6, containing 6% GP; (3) GP12, containing 12% GP; and (4) GP18, containing 18% GP (DM basis). In this experiment, the Siah Gohar grape variety was selected due to its widespread availability in Khorasan Razavi, Iran, where the study was conducted. GP from this variety was collected from local orchards, dewatered, and dried before incorporation into the diets. The isocaloric [containing 2.8 Mcal/kg metabolisable energy (ME)] and isonitrogenous [containing 14 g/kg crude protein (CP)] diets consisting of 30% forage and 70% concentrate were formulated to meet the energy and protein requirements of growing lambs using the Small Ruminant Nutrition System (SRNS) (Cannas et al. 2010). The ingredients and chemical composition of diets and FA profile and phenolic compounds of GP are given in Tables 1 and 2, respectively. Throughout the experiment, animals received ad libitum total mixed ration (TMR) twice daily (7:30 and 17:30), ensuring a daily refusal margin of 10% with free access to the clean water. Individual dry matter intake (DMI) was calculated using daily feed offered and refuse averaged over the interval of the trial. All lambs were weighed individually after an overnight fast before the morning feeding at the start and end of the trial using a calibrated scale (ASA2200, Sepahan Towzin Co., Isfahan, Iran) to determine the ADG. Feed to gain (F:G) ratio was calculated as DMI (kg/d) / ADG (kg/d).

**TABLE 1:** Ingredient and chemical composition of experimental diets.

	Diets^a^			
Item	Ctrl	GP6	GP12	GP18
**Ingredients, %**				
Alfalfa hay	20.0	20.0	20.0	20.0
Wheat straw	10.0	10.0	10.0	10.0
Barley	20.0	20.0	20.0	20.0
Corn	15.0	15.0	15.0	15.0
Soybean Meal	8.00	8.00	8.00	8.00
Wheat bran	6.75	6.75	6.75	6.75
Beet Pulp	18.0	12.0	.00 6	0.00
GP	0.00	6.00	12.0	18.0
Salt	0.25	0.25	0.25	0.25
Calcium carbonate	0.500	0.500	0.500	0.500
Bicarbonate Sodium	0.500	0.500	0.500	0.500
Vitamin‐mineral mix^b^	1.00	1.00	1.00	1.00
**Chemical composition, % of DM**				
Dry Matter	88.0	88.0	88.0	88.0
ME (Mcal/kg)	2.80	2.84	2.88	2.92
CP	14.0	14.0	14.0	14.0
Calcium	0.870	0.880	0.900	0.910
Phosphorus	0.320	0.320	0.330	0.330
NDF	36.7	35.6	34.5	33.45
ADF	19.6	19.6	19.6	19.6
EE	2.25	2.56	2.88	3.21
Total phenols	0.580	1.73	2.77	3.90
Total tannins	0.230	0.960	1.67	2.40

Abbreviations: GP = Grape pomace.

^a^ Diets consisting a control with no GP (Ctrl) and three groups with GP as a substitute for beet pulp at varying levels including 6%, 12%, and 18% DM of diets.

^b^ Contained (/kg of premix; DM basis): 330,000 IU of vitamin A, 60,000 IU of vitamin D, 1000 IU of vitamin E, 160 g Ca, 85 g P, 63 g Na, 45 g Mg, 2100 mg Zn, 1500 mg Mn, 535 mg Cu, 12 mg Se, 45 mg I.

**TABLE 2:** Chemical composition, fatty acid composition, and total phenolic compounds of grape pomace (GP).

Item	GP
DM, %	91.80
CP, % of DM	9.43
EE, % of DM	6.42
ADF, % of DM	21.90
NDF, % of DM	29.95
Ash, % of DM	4.26
Ca, % of DM	1.16
P, % of DM	0.226
Mg, % of DM	0.149
Fe, ppm	116.7
WSC, % of DM	27.05
TP, % of DM	18.072
TT, % of DM	11.616
**Fatty acid, g/100 g of total fatty acids**	
C12:0	0.03
C14:0	0.08
C16:0	7.84
cis9‐C16:1	0.56
C17:0	0.10
C18:0	2.27
cis9‐18:1	17.38
cis6‐18:2	69.89
cis3‐18:3	0.26
C20:0	0.43
C20:1	0.16
SFA	10.75
MUFA	18.10
PUFA	71.15

Abbreviations: ADF, acid detergent fibre; Ca, calcium; CP, crude protein; DM, dry matter; EE, ether extract; FE, ferrum (iron); Mg, magnesium; MUFA, monounsaturated fatty acids; NDF, neutral detergent fibre; P, phosphorus; PUFA, polyunsaturated fatty acids; SFA, saturated fatty acids.

Rumen fluid samples were collected from each lamb using a stomach tube attached to a vacuum pump 2 h after the morning feeding on day 90. The rumen fluid samples were filtered through four layers of cheesecloth and immediately used to measure its pH using a glass electrode pH meter (691 Metrohm, Herisau, Switzerland). The ruminal fluid was subsequently acidified with 10 mL of 0.2 N HCl solution (50%, vol/vol) and stored frozen at ‐20°C before ammonia‐N analysis.

Individual blood samples were collected into sterile tubes containing EDTA solution from the jugular vein 3 h after the morning feeding (10 mL/lamb) at the end of the experiment. Plasma samples were obtained by centrifuging the blood tubes for 15 min at 3,000 × g and then were frozen at −20°C until analysis.

At the end of a 90‐day trial period, lambs were electrically stunned and slaughtered. Carcasses were immediately transferred to a cooler at 4°C, and after a conservation period of 24 h, samples of *Longissimus lumborum* (LL) muscles (between the 12th thoracic and 5th lumbar vertebrae), muscle and subcutaneous adipose tissue adjacent to the right half carcass were obtained, vacuum packaged, and stored at −20°C until analysis.

### 2.3 Laboratory Analysis

For chemical analyses, the samples of feed and orts were milled to pass through a 1 mm screen by a Cyclotec^TM^ 1093 Sample Mill (Foss Companies, Hillerød, Denmark). Samples were analysed for DM (method 934.01; AOAC 1990), organic matter (method 920.39; AOAC 1990), ether extract (method 920.39; AOAC 1990) and CP (method 988.05; AOAC 1990) standard procedures. Concentrations of acid detergent fibre (ADF) inclusive of residual ash (method 973.18; AOAC 1990) and neutral detergent fibre (NDF) inclusive of residual ash were determined sequentially without the use of sodium sulphite and with the inclusion of α‐amylase (Van Soest et al. 1991). For the tannin assay, GP samples were dried at 40°C and ground to 0.5 mm. Phenolic compounds were extracted from 200 mg of the sample using 70% aqueous acetone, left overnight at 4°C. Extracts were centrifuged at 3000 g for 15 min at 4°C. The concentration of total phenolic compounds (TP) was determined using the Folin–Ciocalteu procedure, and total tannins (TT) were estimated indirectly after being absorbed to insoluble polyvinylpolypyrrolidone (PVPP) (Makkar 2000).

The concentrations of glucose (Glu), cholesterol (CHOL), triglyceride (TG), blood urea nitrogen (BUN), total protein (TP), albumin (ALB), and liver enzymes, including alanine aminotransferase (ALT) and aspartate aminotransferase (AST), were determined using commercial kits (Pars Azmoon Company, Tehran, Iran) and an automated biochemistry analyser (Midrary BS800M, Shenzhen, China) according to the manufacturer's instructions.

The FA composition of muscle tissue was determined following the extraction of total lipids using the method described by Folch et al. (1957). Briefly, samples (0.5 g each, with two replicates) were placed in 5 mL screw‐top test tubes, and 2 mL of 2 N methanolic potassium hydroxide were added. The tubes were then sealed and vigorously shaken for 2 s. Subsequently, 2 mL of hexane were added to the tubes, which were shaken for 5 s before being immersed in an ultrasonic bath at 35°C for 15 min. Afterward, the upper layer was separated and filtered through a 0.45 mm filter containing anhydrous sodium sulphate. Finally, 1 mL of the resulting filtrate was injected into a gas chromatograph (YL6100 GC, Young Lin Instrument, Anyang, Korea). The gas chromatograph was equipped with a J&W CP‐Sil 88 fused silica capillary column (100 m × 0.25 mm, 0.20 mm film thickness, Agilent Technologies, USA). The temperatures of the injector and detector ports were set at 270°C and 300°C, respectively. The fatty acid composition was analysed using an isothermal program, with the column temperature maintained at 175°C for 60 min. Individual fatty acid methyl esters (FAME) were identified based on a standard mixture of 37 Component FAME Mix (FAME Mix C4‐24, 18919‐1AMP, Supelco, Sigma‐Aldrich, Bellefonte, PA, USA) and 60 individual FAME standards (Sigma‐Aldrich, USA). The identification of conjugated linoleic acid (CLA) isomers was facilitated by co‐injection with commercial standard mixtures (Sigma Aldrich, Bellefonte, PA, USA). The concentration of fatty acids was expressed as grams per 100 grams of total FAME.

Lipid oxidation of muscle was measured by the 2‐thiobarbituric acid distillation procedure modified by Buege and Aust (1978) assay of lipid oxidation in muscle samples. Results were expressed as 2‐thiobarbituric acid reactive substances (TBARS) in mg of malondialdehyde (MDA)/kg of muscle. TBARS values were measured in duplicate on days 0, 7, 14, and 21 for meat samples.

### 2.4 Statistical Analysis

Data were statistically analysed as a completely randomised design (CRD) using the SAS software (SAS Institute, version 9.0; SAS Institute Inc., Cary, NC, USA). Data distribution and homogeneity of variances were tested using the UNIVARIATE procedure, confirming that all variables met normality (Shapiro‐Wilk test, p > 0.05) and homogeneity of variances (Levene's test, p > 0.05) assumptions. Performance data, collected over multiple time points, were analysed using the MIXED procedure for repeated measures because it accounts for the correlation structure of longitudinal data and accommodates random effects, such as individual lamb variability, ensuring robust analysis of time‐dependent responses (Littell et al. 1998). The fixed effects in the model were diets, time, and their interaction (diet × time), while lambs were nested as a random factor. The IBW of lambs was also used as a covariate in the model. Performance data was subjected to covariance structures (autoregressive 1, spatial power, and unstructured), and the one resulting in the lowest Akaike information criterion was chosen.

While once‐time measured data (rumen fermentation, blood parameters, and meat FA profile) was analysed using the GLM procedure with the following statistical model:

$$Y_{\mathrm{ij}}=\mu +A_{i}+e_{\mathrm{ij}}$$

where *Y*_ij_ is the measured value, μ is the overall mean of the population, A_i_ is the treatment, and e_ij_ is the error term.

Least‐square means (LSM) were computed and tested for differences using the Tukey–Kramer test. The significance level was set at *p* < 0.05, while *p* ≤ 0.10 was considered as a tendency.

## 3 Results and Discussion

### 3.1 Growth Performance

Dietary inclusion of GP did not affect the DMI (*p* = 0.8452) in fattening lambs (Table 3). While the IBW was similar (*p* = 0.186) across groups, the highest (*p* = 0.027) final body weight (FBW) was for GP18, followed by GP12. The ADG of lambs in the GP12 and GP16 groups were 5.8% and 8.1% higher (*p* = 0.016) than in the Ctrl group, respectively. Additionally, lambs fed on GP12 and GP16 diets showed the lowest (*p* = 0.001) feed‐to‐gain ratio (F:G).

**TABLE 3:** Fattening performance from Baluchi male lambs fed with different grape pomace (GP) levels.

Item	Diets^d^				SEM^e^	*p*‐value
	Ctrl	GP6	GP12	GP18		
IBW, kg	28.50	27.76	28.44	28.68	0.899	0.1864
FBW, kg	50.82^b^	50.72^b^	52.04^ab^	52.82^a^	1.519	0.0270
DMI, kg	1.687	1.724	1.700	1.702	0.088	0.8421
ADG, kg	0.223^c^	0.230^bc^	0.236^ab^	0.241^a^	0.110	0.0167
F:G ratio	7.56^a^	7.51^a^	7.21^b^	7.05^b^	0.2018	0.0001

*Note*: Values are least‐square means.

Abbreviations: ADG, average daily gain; DMI, dry matter intake; FBW, final body weight; F:G, feed to gain ratio; IBW, initial body weight.

^abc^The values within a row with different superscripts are significantly different (p ≤ 0.05); the highest values are indicated with the letter ‘a’, followed by ‘b’ and ‘c’.

^d^ Ctrl, GP6, GP12, and GP18 contained 0%, 6%, 12%, and 18% grape pomace on a DM basis, respectively.

^e^ Pooled standard error of the mean.

The unaffected DMI suggests that GP inclusion did not compromise palatability or consumption, consistent with studies reporting no changes in nutrient intake following GP supplementation in lambs (Massaro et al. 2021; Zhao et al. 2018; Boran et al. 2025), heifers (Ream et al. 2021), and steers (Molosse et al. 2023). In confirmation, Chikwanha et al. (2019) found that GP in finisher lambs up to 20% DM of diet did not induce bitter and astringent sensations associated with the consumption of proanthocyanidins, which bind to salivary glycoproteins and potentially cause adverse post‐ingestive effects. Supplementation of grape seed oil (up to 4% DM of diet) is also reported to have no effect on DMI in fattening lambs (Sharifi et al. 2019). However, it is worth noting that the effects of GP on DMI mainly depend on the inclusion level, residue profile, and animal species. For lambs, GP levels exceeding 20% can reduce DMI due to elevated phenolic and lignin contents (Calderón‐Cortés et al., 2018). In the present trial, the diets were isoenergetic and isonitrogenous, and GP inclusion was set lower than the critical level reported in the literature, which likely explains the lack of significant effects on DMI.

The improved ADG and F:G align with previous studies on GP application in feedlot lambs (Chikwanha et al., 2019a; Kafantaris et al. 2018). In confirmation, Chikwanha (2019 b) demonstrated that the moderate to low levels of proanthocyanidins in GP may bind to dietary proteins or microbial enzymes in the rumen and/or inhibit the growth of microbes involved in protein degradation, leading to an increased flow of essential amino acids to the duodenum and potentially improving growth performance. In line with our results, Zhao et al. (2018) found greater ADG and lower F:G ratios with 5% and 10 % GP inclusion in lamb diets, indicating improved feed efficiency. Similarly, lambs fed 8% GP exhibited higher FBW and carcass yields (Yao et al. 2023), and Angus bulls with 10% GP exhibited improved ADG and fattening performance (Li et al. 2024). These benefits likely stem from moderate polyphenol levels improving protein utilisation by binding dietary proteins (Frutos et al. 2004) or inhibiting ruminal microbial degradation, thus increasing amino acid flow to the small intestine (Toral et al. 2011). Furthermore, GP's antioxidant properties, including reactive oxygen species (ROS) scavenging, may reduce intestinal membrane damage, enhancing gut functionality and supporting growth (Kafantaris et al. 2018). In confirmation, Kafantaris et al. (2017) demonstrated that the GP could favour growth performance due to its antioxidant properties. Collectively, these results highlight GP's potential as a sustainable feed ingredient that enhances lamb growth efficiency, supporting its role in reducing reliance on conventional feeds and promoting circular economy practices.

### 3.2 Ruminal Fermentation Characteristics

Dietary inclusion of GP tended to increase ruminal pH (*p* = 0.100) in fattening lambs; however, NH3‐N levels remained unaffected (*p* = 0.160) (Table 4). The GP6 group exhibited the highest concentrations of acetate (*p* = 0.003) and valerate (*p* = 0.001), while GP18 showed the highest propionate levels (*p* = 0.003). Lambs fed GP6 and GP12 had higher butyrate concentrations (*p* = 0.003) than the Ctrl. There were no diet effects on isobutyrate (*p* = 0.726) and isovalerate (*p* = 0.156).

**TABLE 4:** Ruminal fermentation parameters from Baluchi male lambs fed with different grape pomace (GP) levels.

Item	Diets^d^				SEM^e^	*p*‐value
	Ctrl	GP6	GP12	GP18		
**Ruminal parameters**						
pH	6.64	6.44	6.70	6.90	0.3753	0.1099
NH3‐N (mg/dl)	3.15	3.64	4.89	4.03	1.623	0.1695
**VFA (mmol)**						
Acetate	57.65^b^	63.42^a^	56.46^b^	57.63^b^	3.055	0.0031
Propionate	30.06^a^	30.43^a^	23.58^b^	31.74^a^	2.432	0.0002
Butyrate	8.75^b^	11.95^a^	12.56^a^	4.70^c^	2.463	0.0003
Iso butyrate	0.797	0.697	0.677	0.610	0.3769	0.7264
Valerate	1.183^bc^	2.170^a^	1.5233^b^	0.927^c^	0.4582	0.0013
Iso valerate	0.963	1.233	1.497	0.883	0.6016	0.1561

*Note*: Values are least‐square means.

^abc^ The values within a row with different superscripts are significantly different (p ≤ 0.05); the highest values are indicated with the letter ‘a’, followed by ‘b’ and ‘c’.

^d^ Ctrl, GP6, GP12, and GP18 contained 0%, 6%, 12%, and 18% grape pomace on a DM basis, respectively.

^e^ Pooled standard error of the mean.

Ruminal pH values fell within the optimum ranges (6.7 ± 0.5) for cellulolytic bacteria activity, supporting efficient rumen microbial function and nutrient digestion (Aschenbach et al. 2011). This aligns with Vinyard et al. (2021), who reported a quadratic increase in maximum pH with GP inclusion. In addition, ruminal NH_3_‐N concentration were found to surpass the minimum threshold of 0.85 mg/L required for microbial growth (Aschenbach et al. 2011). Although dietary GP inclusion was expected to decrease ruminal NH_3_‐N concentrations due to polyphenolic compounds affecting nitrogen metabolism (Sharifi et al. 2019), no changes were observed. This finding is consistent with studies reporting stable NH_3_‐N levels following GP inclusion up to 30% DM in lambs (Vinyart et al. 2021), and 7.5% to 27% in dairy cows (Moate et al. 2014; Pauletto et al. 2020). However, conflicting results, such as decreased (Chikwanha et al. 2019) or increased (Correddu et al. 2015) NH_3_‐N levels likely attributed to variations in GP composition (e.g., tannin levels and drying processing), dietary matrix effects, animal‐specific factors (e.g., breed and rumen microbial populations), and analytical methods (Moate et al. 2014; Pauletto et al. 2020). In our study, condensed tannin (CT) levels below 50 g/kg DM and drying GP likely minimised polyphenolic activity, limiting tannin effects on proteolytic microbes and NH_3_‐N levels (Kelln et al. 2020).

Elevated acetate and propionate in GP6 and GP18 align with findings from tannin‐rich diets in dairy sheep (Toral et al. 2011; Correddu et al. 2015). As a confirmation, Cheng et al. (2023) showed that the dietary supplement of 8% GP alters the rumen microbiome to promote propionate production. Increased butyrate with 6% and 12% GP inclusion levels was likely driven by its interconversion with acetate via intermediate substrates, supporting the anabolic activity of anaerobic bacteria (Hackmann and Firkins 2015). High butyrate‐producing bacteria, such as the *Butyrivibrio fibrisolvens*, may also have contributed, as observed in tannin‐fed animals (Buccioni et al. 2012). Conversely, reduced butyrate in GP18 agrees with Correddu et al. (2015). Similarly, Khiaosa‐Ard et al. (2023) showed a high dosage of GP (20% DM of diet) increased acetate at the expense of butyrate.

The lower SCFA profile GP18‐fed lambs mirror results from steers fed 10% of GP silage and GP bran (Molosse et al. 2023). Also, similar subtle modulations have been reported by Moate et al. (2014) and Vinyard et al. (2021). These effects likely reflect tannins's influence on microbial fermentation (Vasta et al. 2019), as seen with high GP levels (30% DM) in beef cattle (Caetano et al. 2019). The inclusion of GP has been linked to increased acetate (Moate et al. 2014), unchanged propionate (Moate et al. 2014), and even decreased propionate (Tayengwa and Mapiye, 2018); these variations may arise from the processing of the GP components (Molosse et al. 2023). It is worth noting that SCFA production is influenced by diet composition, ingredient quality, and additives (Aschenbach et al. 2011), which may explain minor variations in SCFA modulation observed with GP inclusion. Collectively, these results highlight that a GP's impact on ruminal fermentation depends on a complex interplay of tannin concentration, dietary adaptation, and individual animal variation. By modulating VFA profiles, particularly acetate and propionate, GP offers potential to optimise energy availability in lambs, supporting its use as a sustainable feed ingredient. However, standardised processing is critical to minimise variability and enhance practical applications in ruminant diets.

### 3.3 Blood Metabolites

Concentrations of glucose (*p* = 0.508), triglyceride (*p* = 0.966), cholesterol (*p* = 0.449), albumin (*p* = 0.945), creatinine (*p* = 0.928), and BUN (*p* = 0.659) were not affected by the diets. However, GBP6 increased (*p* = 0.049) total protein concentration compared to the Ctrl (Table 5). No significant differences were observed in aspartate aminotransferase (AST; *p* = 0.926) and alanine aminotransferase (ALT; *p* = 0.981) concentrations.

**TABLE 5:** Blood metabolites from Baluchi male lambs fed with different grape pomace (GP) levels.

Item	Diets^c^				SEM^d^	*p*‐value
	Ctrl	GP6	GP12	GP18		
Glucose (mg/dl)	60.67	57.00	61.83	63.33	41.89	0.5080
Triglyceride (mg/dl)	18.67	20.00	21.67	17.67	19.34	0.9661
Cholesterol (mg/dl)	50.33	59.67	65.00	60.00	20.16	0.4493
Albumin (g/dl)	3.20	3.27	3.27	3.20	0.360	0.9450
Creatinine (mg/dl)	1.13	1.17	1.23	1.20	0.364	0.9280
Total protein (g/dl)	6.47^b^	7.90^a^	7.07^ab^	7.00^ab^	0.954	0.0491
BUN (mg/dl)	44.00	39.00	39.33	40.67	9.990	0.6590
AST (U/L)	140.0	128.3	126.0	132.0	51.65	0.9269
ALT (U/L)	29.67	28.67	31.33	30.00	15.30	0.9818

*Note*: Values are least‐square means.

Abbreviations: ALT, alanine aminotransferase; AST, aspartate aminotransferase; BUN, blood urea nitrogen.

^ab^ The values within a row with different superscripts are significantly different (p ≤ 0.05); the highest values are indicated with the letter ‘a’ followed by ‘b’.

^c^ Ctrl, GP6, GP12, and GP18 contained 0%, 6%, 12%, and 18% grape pomace on a DM basis, respectively.

^d^ Pooled standard error of the mean.

These findings align with previous research showing that dietary inclusion of 2% and 4% grape seed oil also did not alter plasma concentrations of glucose, albumin and total protein in fattening lambs (Sharifi et al. 2019). Similarly, a diet containing 15% GP had no significant effect on the serum levels of α‐lactalbumin, caseins, and ALB in dairy cows (Chedea et al., 2017). Increased total protein in the GP6 group may reflect enhanced protein utilisation, possibly due to GP's polyphenolic compounds protecting dietary proteins (Toral et al. 2011). AST and ALT, liver‐specific enzymes released during hepatocellular damage (Mahgoub et al., 2008), showed no changes, indicating that GP inclusion up to 18% DM does not induce hepatotoxicity in Baluchi lamb. This supports the safety of GP as a feed ingredient, consistent with Molosse et al. (2023), who reported unchanged plasma levels of albumin, glucose, urea, and gamma‐glutamyl transferase in steers fed GP silage and bran. The stable blood metabolite profiles suggest that GP maintains metabolic homeostasis, reinforcing its potential for practical use in commercial lamb feeding programmes and contributing to sustainable ruminant nutrition strategies.

### 3.4 Meat Fatty Acid Profile

Meat SFA content increased (*p <* 0.0001) with higher GP supplementation (Table 6). The MUFA (*p <* 0.0001) was higher in the GP8 and Ctrl groups than that for GP12 and GP18. While the highest PUFA (*p <* 0.0001) levels were observed in meat from lambs fed GP18. The GP18 increased C18:3 n‐3 (LNA, *p =* 0.005), C18:2 cis‐6 (LA, *p < 0*.0001), and C18:0 (SA, *p < 0.0001*) contents. The 18:0 content was highest in the GBP18 group, while cis‐9 18:1 (OA) decreased (*p <* 0.0001). The concentration of C12:0, C13:0, C14:0, C15:0, C16:0, trans‐9 18:1, and C20:0 also increased in GP‐fed groups compared to the Ctrl diet (*p <* 0.0001). Likewise, C20:3 n‐6 (*p <* 0.0001) rose in the meat with increasing GP inclusion, while C20:3 n‐3 increased significantly only in the GP18 group (*p* < 0.001) compared to Ctrl. It is important to note that the concentrations of C12:0 and C13:0 should be approached with caution as they were near or at the limit of detection in our study and may not be fully quantitative. However, these FAs had negligible impact on the overall conclusions drawn from the study.

**TABLE 6:** Fatty acid composition (g/100 g FAME) of *longissimus lumborum* (LL) muscle from Baluchi male lambs fed with different grape pomace (GP) levels.

Fatty acid, g/100 g of FA	Diets^c^				SEM^d^	*P*‐Value
	Ctrl	GP6	GP12	GP18		
C10:0	0.00^b^	0.1167^a^	0.1133^a^	0.00^b^	0.0243	<0.0001
C12:0	0.00^c^	0.1067^b^	0.150^a^	0.00^c^	0.0237	<0.0001
C13:0	0.00^b^	0.00^b^	0.023^a^	0.00^b^	0.0054	<0.0001
C14:0	2.163^d^	2.583^b^	3.490^a^	2.293^c^	0.0804	<0.0001
C14:1	0.187^b^	0.117^c^	0.133^c^	0.287^a^	0.0303	<0.0001
C15:0	0.55^c^	0.6^3b^	0.80^a^	0.57^c^	0.0575	<0.0001
C16:0	22.84^c^	23.51^b^	26.50^a^	26.35^a^	0.4014	<0.0001
C16:1	1.67^b^	1.69^b^	2.18^a^	1.38^c^	0.0965	<0.0001
C17:0	2.37^a^	2.04^bc^	1.95^c^	2.13^b^	0.1008	<0.0001
C18:0 (SA)	19.11^c^	18.70^d^	19.48^b^	25.09^a^	0.2002	<0.0001
C18:1 trans‐9	0.63^c^	1.160^a^	1.087^a^	0.90^b^	0.0875	<0.0001
C18:1 cis‐9 (OA)	44.53^a^	43.98^b^	39.47^c^	33.59^d^	0.4786	<0.0001
C18:2 cis‐6 (LA)	4.39^b^	3.81^c^	3.29^d^	4.86^a^	0.0919	<0.0001
C20:0	0.00^d^	0.120^c^	0.177^b^	0.30a	0.0403	<0.0001
C20:1 n‐9	0.33^b^	0.41^ab^	0.45^a^	0.41^ab^	0.0808	0.0544
C18:3 n‐3 (LNA)	0.117^b^	0.113^b^	0.11^b^	0.1667^a^	0.0282	0.0050
C22:0	0.00^b^	0.00^b^	0.027^a^	0.00^b^	0.0054	<0.0001
C20:3 n‐3	1.00^b^	0.65^c^	0.21^d^	1.22^a^	0.0786	<0.0001
C18:3 n‐6	0.00^b^	0.023^a^	0.027^a^	0.00^b^	0.0077	<0.0001
C18:2 trans‐6	0.00^c^	0.00^c^	0.19^b^	0.23^a^	0.0272	<0.0001
C20:3 n‐6	0.00^d^	0.087^b^	0.030^c^	0.130^a^	0.0255	<0.0001
SFA	47.03^d^	47.81^c^	52.70^b^	56.73^a^	0.4661	<0.0001
MUFA	47.35^a^	47.36^a^	43.32^b^	36.57^c^	0.5303	<0.0001
PUFA	5.51^b^	4.68^c^	3.85^d^	6.61^a^	0.1301	<0.0001
TFA	0.63^c^	1.16^b^	1.27^a^	1.13^b^	0.0833	<0.0001
SCFA	0.00^b^	0.12^a^	0.11^a^	0.00^b^	0.0243	<0.0001
MCFA	27.41^d^	28.63^c^	33.27^a^	30.88^b^	0.3640	<0.0001
LCFA	72.48^a^	71.10^b^	66.49^d^	69.03^c^	0.4548	<0.0001

*Note*: Values are least‐square means.

Abbreviation: LCFA, long‐chain fatty acids; MCFA, medium‐chain fatty acids; MUFA, monounsaturated fatty acids; PUFA, polyunsaturated fatty acids; SCFA, short‐chain fatty acids; SFA, saturated fatty acids; TFA, trans fatty acids.

^ab^ The values within a row with different superscripts are significantly different (*p* ≤ 0.05); the highest values are indicated with the letter ‘a’, followed by ‘b’ and ‘c’.

^c^ Ctrl, GP6, GP12, and GP18 contained 0%, 6%, 12%, and 18% grape pomace on a DM basis, respectively.

^d^ Pooled standard error of mean.

Consumers increasingly prioritise meat FA composition due to its links to health outcomes, such as cardiovascular disease and cancer (Ponnampalam et al. 2024). Feeding strategies aim to reduce SFA (e.g., C16:0) and specific trans‐FAs (e.g., C18:1 t‐10) while boosting conjugated linoleic acid (CLA) isomers (e.g., C18:2 c9t11), certain trans‐FA isomers (e.g., C18:1 t‐11), and n‐3 and n‐6 PUFAs (e.g., C18:3 n‐3, C18:2 n‐6) to enhance meat's health value (Ponnampalam et al. 2024). Dietary lipids, FAs, and polyphenolic compounds are key factors in manipulating ruminant FA profiles, despite limited modification capacity (Vasta et al. 2019).

GP's high linoleic acid (18:2 n‐6) content and polyphenolic compounds likely reduced ruminal biohydrogenation, allowing greater PUFA deposition in meat (Siller‐Sánchez et al. 2024; Vasta et al. 2019). Polyphenols may inhibit biohydrogenation by limiting the growth and activity of microbes, such as Butyrivibrio species, thereby increasing PUFA ruminal escape (Vasta et al. 2019). Notably, GP is rich in alpha‐linolenic acid (18:3n‐3, ALA), a precursor to health‐beneficial eicosapentaenoic (20:5n‐3, EPA) and docosahexaenoic (22:6n‐3, DHA) acids (Alfaia et al., 2022). The elevated PUFA levels (including C18:3 n‐6, C20:1 n‐9, C20:3 n‐3, C20:3 n‐6) in GP‐fed lambs likely resulted from increased C18:2 n‐6 undergoing elongation and desaturation (Lee et al., 2016). These findings align with Arend et al. (2022), who reported a 58 to 80% increase in the 18:2 n‐6 in muscle from GP‐fed Steers and Sharifi et al. (2019), who noted improved C18:2 n‐6, PUFA, and n‐6:n‐3 ratio in lamb intramuscular fat with grape seed oil supplementation. Similarly, Akter et al. (2025) found that high linoleic acid in GP contributes to the increased levels of 18:2 n‐6 in the milk fat of cows fed 10–15% GP (DM basis).

The increased PUFA profiles in the current study agree with polyphenol‐rich diets in ruminants (Vasta et al. 2019; Sharifi et al. 2019). In line with our results, a recent review (Alfaia et al., 2022) with monogastrics demonstrated that GP enhanced LA, LNA, PUFA, n‐3 PUFA, and PUFA/SFA ratio in pork and poultry meat. In ruminants, polyphenols in GP likely reduced ruminal lipolysis and protected PUFA from biohydrogenation by altering microbial cell walls, substrate availability, or membrane permeability (Mueller‐Harvey et al., 2019). However, higher PUFA levels in GP‐fed lambs corresponded with reduced C18:1 n‐9, likely due to PUFA inhibiting delta‐9 desaturase activity, as reported by Ponnampalam et al. (2014) and Vasta et al. (2019). This inverse relationship is supported by Siller‐Sánchez et al. (2024), Arend et al. (2022), and Sharifi et al. (2019). The Ctrl diet may have favoured de novo FA synthesis, increasing C18:1 n‐9 via elongation and desaturation of C16:0 and C18:0 (Vasta et al. 2019).

Taken together, these findings indicate that supplementing lamb feed with GP can promote incomplete biohydrogenation and positively influence the FA profile of meat. Increases in PUFA, including C18:3 n‐3 and C18:2 cis‐6, suggest a potential improvement in the health benefits of meat fat. However, the rise in palmitic acid may counteract these beneficial effects, and higher concentrations of n‐3 and n‐6 PUFA may increase the susceptibility of meat to oxidative spoilage.

### 3.5 Meat Antioxidant Stability

**FIGURE 1:**
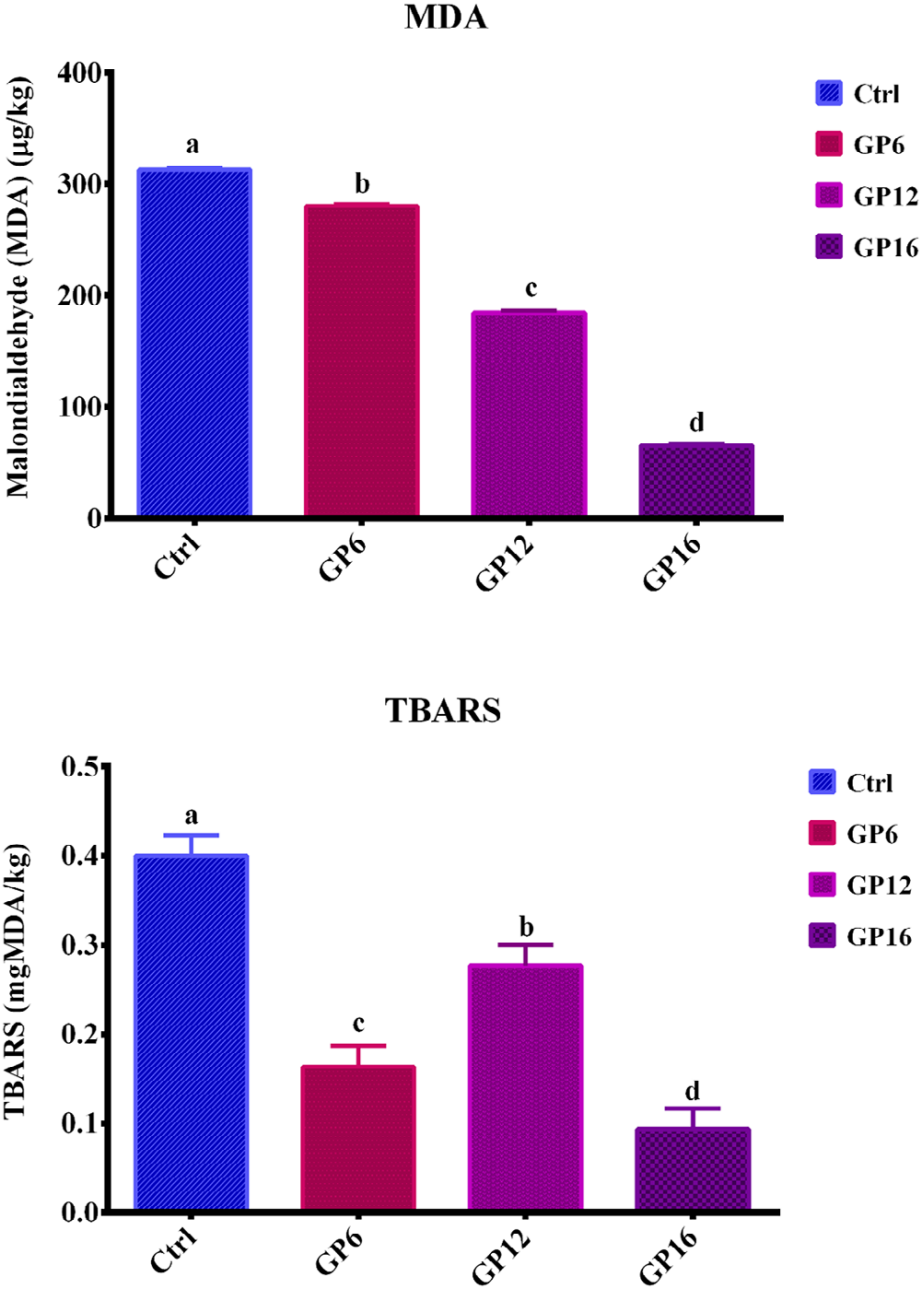
Lipid oxidation indicators of *longissimus lumborum* (LL) muscle from Baluchi male lambs fed with different grape pomace (GP) levels.

Dietary inclusion on GP mitigated MDA (*p < 0.0001*) levels in lamb's meat, with GP6, GP12, and GP18 groups showing 10.9%, 41.3%, and 82.1% lower MDA levels than the Ctrl group, respectively (Figure 1). The TBARS levels also decreased (*p < 0.0001*) with increasing GP inclusion, being the lowest values for GP18.

Lipid oxidation, driven by ROS, is the primary non‐microbial cause of meat deterioration (Lund et al. 2011). Antioxidant feeding strategies that promote cellular incorporation are thus critical for enhancing meat oxidative stability (Vasta et al. 2019). Despite the higher PUFA content in GP‐fed lambs, which could increase oxidative susceptibility, the substantial reduction in MDA levels highlights the potent antioxidant properties of GP's polyphenolic compounds. These findings suggest that GP can simultaneously improve meat's nutritional quality and extend shelf life, offering significant benefits for the meat industry. It has been well established that the proanthocyanidin present in GP exhibits strong scavenging against superoxide anion, hydroxyl and peroxyl radicals (Bagchi et al. 1997), positioning GP as a promising feed ingredient for improving the oxidative stability of lambs.

These results align with previous studies, such as Arend et al. (2022), who reported reduced MDA content in beef from steers fed 58% GP (dry matter, DM) and Chikwanha et al. (2019), who observed the lowest MDA levels in lamb meat after five days of storage with 20% GP inclusion. Zhao et al. (2018) suggested that GP supplementation (5% and 10 % DM) increases Nrf2 mRNA expression in lambs, activating antioxidant response elements in genes encoding antioxidative enzymes. Similarly, Sharifi et al. (2019) found that dietary addition of grape seed oil (2% and 4% of DM) improved antioxidant levels and reduced susceptibility to lipid oxidation in lambs *longissimus lumborum* (LL) muscle as measured by the reaction of 2‐thiobarbituric acid with MDA. These improvements are likely attributed to the transfer and incorporation of dietary polyphenols and their metabolites into muscle tissue, where they function as cellular antioxidants. This process represents a natural biofortification approach that enhances meat quality without synthetic additives (Vastolo et al. 2024; Correddu et al., 2023).

Polyphenols, including those in GP, reduce lipid and protein oxidation by directly neutralising ketones, aldehydes, and alcohols or by enhancing natural antioxidant defences (Li et al. 2024; Kafantaris et al. 2017; Vasta et al. 2019). They may also limit lipid oxidation by inhibiting the release of C20:4 n‐6 from phospholipids (Correddu et al. 2023). Zhao et al. (2018) reported that GP inclusion (5% and 10% DM) increased total antioxidant capacity (TAC) and superoxide dismutase (SOD) activity in lamb meat. Although muscle phenolic content was not measured in this study, feeding the GP diet likely increased these levels, thereby improving antioxidative status. This supports the inclusion of GP as a beneficial strategy in the lamb industry to extend the shelf life of beef products, addressing concerns about synthetic antioxidants.

### 3.6 Implications and Public Dissemination

GP at 6–18% DM enhances lamb growth (ADG up 3.1–8.1%, higher FBW) without affecting DMI, optimises ruminal fermentation (increased VFA, pH), and improves meat quality with more PUFAs (e.g., C18:3 n‐3) and less lipid oxidation (MDA down 10.9–82.1%). These findings have significant practical implications for sheep farming under operational conditions. GP, a winery by‐product, can replace conventional feed ingredients like beet pulp, reducing costs and environmental waste while maintaining or improving lamb performance and meat quality (Polo et al. 2023; Vastolo et al. 2024, Vasta et al. 2019). Its antioxidant properties extend meat shelf life, addressing consumer demands for natural, healthier products and supporting sustainable production systems (Akter et al. 2025; Zhao et al. 2018). However, standardised processing is critical to minimise variability and enhance practical applications in lamb diet. Also, to maximise the impact of these results, bridge the gap between scientific research in ruminant nutrition and public knowledge; disseminating our findings through social media platforms such as Twitter, Facebook, and Instagram is essential, as these platforms foster the engagement of farmers and consumers and effectively convey complex topics in ruminant nutrition (Lamanna et al. 2025).

## 4 Conclusion

Dietary inclusion of GP at 6%, 12%, and 18% DM did not affect the DMI, while enhancing FBW in fattening lambs. The ADG increased by 5.8% and 8.1% in the GP12 and GP18 groups, respectively, compared to the Ctrl group. Also, GP tended to elevate ruminal pH, while NH_3_‐N levels remained unaffected. Furthermore, GP ingestion, particularly at an inclusion level of 18% DM, increased muscle accumulation of health‐promoting FA, including 18:2 n‐6, 18:2 c9t11, C18:3 n‐3, C20:3 n‐3, and total PUFA, likely due to polyphenol‐mediated alterations in ruminal biohydrogenation pathways. Additionally, GP mitigated lipid oxidation in lamb meat, as indicated by 10.9% 41.3%, and 82.1% lower MDA levels in the GP6, GP12, and GP18 groups, respectively. Further investigation is warranted to verify the potential effects of GP on the rumen microbiome, meat colour stability, and shelf life, as well as to elucidate the mechanisms underlying the action of its polyphenols.

## Author Contributions

**Moslem Bashtani**: conceptualisation, project administration, methodology, supervision. **Mahdi Badiee**: conceptualisation, project administration, data curation, formal analysis, funding acquisition, investigation, methodology, farm sampling, software. **Seyyed Homayoun Farhangfar**: data curation, formal analysis, software; **Navid Ghavipanje**: writing ‐ original draft, writing ‐ review and editing, visualisation.

## Ethics Statement

All experimental protocols and implemented procedures were reviewed by the Animal Welfare and Ethical Review Board of the Department of Animal Science, University of Birjand. The authors confirm that the ethical policies of the journal, as noted on the journal's author guidelines page, have been adhered to.

## Conflicts of Interest

The authors declare no conflicts of interest.

## Data Availability

The datasets generated or analysed during this study are included in this published article.
